# Patient experiences of participating in a cancer rehabilitation triage and referral system in the Irish healthcare system

**DOI:** 10.1007/s11764-025-01785-6

**Published:** 2025-03-27

**Authors:** Louise Brennan, Megan Kennedy, Sophie Grehan, Hayley Connolly, Grainne Sheill, Claire L. Donohoe, Emer Guinan

**Affiliations:** 1 Department of Surgery, School of Medicine, Trinity College Dublin, Dublin, Ireland; 2 Trinity St James’s Cancer Institute, Dublin, Ireland; 3 Discipline of Physiotherapy, School of Medicine, Trinity College Dublin, Dublin, Ireland; 4 Discipline of Occupational Therapy, School of Medicine, Trinity College Dublin, Dublin, Ireland; 5 Department of Physiotherapy, St James’s Hospital, Dublin, Ireland; 6 Department of Surgery, St James’s Hospital, Dublin, Ireland; 7 Wellcome – HRB Clinical Research Facility at St. James’s Hospital, Dublin, Ireland

**Keywords:** Exercise, Triage, Referral, Survivorship, Rehabilitation, Physiotherapy, Patient experience

## Abstract

**Background:**

Screening, triage and referral systems can support patients to become more physically active and receive the right level of rehabilitation. This study aimed to understand patient experiences of attending a physiotherapy-led triage and referral clinic.

**Methods:**

Adults who had been diagnosed with cancer during COVID-19 (March 2020 to May 2022) and had completed treatment were invited to attend a physiotherapy-led triage and referral clinic for assessment and triage to one of three needs-based pathways. Participant experiences were captured via semi-structured interviews at 12-weeks post triage assessment. The Consolidated Framework for Implementation Research (CFIR) was used to design interview guides and to guide deductive analysis.

**Results:**

Twenty-six participants (male *n* = 15, mean age 65 (11.6) years, mean 28.5 (6) months post-diagnosis) were interviewed. Key findings were substantial unmet needs and a sense of being unsupported (outer setting), an individual assessment with the specialist physiotherapist was gratefully received (outer setting), cancer rehabilitation services must improve (intervention characteristics), rehabilitation is a worthwhile investment for the health service (inner setting) and ability varies amongst participants to absorb community service costs (intervention characteristics).

**Conclusion:**

Findings indicate high participant satisfaction with personalised assessment and referral to exercise services. The opportunity to meet with a specialist physiotherapist was highly valued by participants across all levels, addressed a significant unmet service need and led to improvements in multiple unmet physical and psychosocial needs.

Implications for Cancer Survivors.

Triage and referral systems have significant potential to support implementation of exercise and rehabilitation pathways into cancer care with high patient satisfaction.

Trial registration.

ClinicalTrials.gov Identifier: NCT05615285.

**Supplementary Information:**

The online version contains supplementary material available at https://doi.org/10.1007/s11764-025-01785-6.

## Introduction

Adverse physical and psychosocial consequences of cancer and cancer treatment which impair function and negatively impact quality of life are commonly reported in people living with and after cancer [1, 2]. These wide-ranging negative side effects include weakness, fatigue, pain, loss of physical fitness, depression, anxiety and poor sleep [3, 4]. It is now well established that exercise, both aerobic and resistance, can help improve many of the negative physical and psychosocial consequences of cancer and improve quality of life, notably physical functioning, cancer-related fatigue and health-related quality of life [5].

The American College of Sports Medicine (ACSM) Exercise Guidelines for Cancer Survivors 2019 recommends that cancer survivors perform a weekly minimum of 150 min of aerobic exercise and two 30-min resistance training sessions, targeting all major muscle groups [5]. However, engaging in physical activity can be challenging for this cohort, and barriers to exercise include lack of knowledge about the benefits of exercise, the impact of treatment side effects, and a lack of specialist professional support to facilitate participation [6]. Exercise services targeted to cancer survivors and existing services are insufficient in number, often under-resourced and unable to fully meet the need of the local population [7]. An implementation analysis of exercise in cancer care found that referral pathways are often fragmented and lack standardisation and suitable expertise [8]. Additionally, it found poor integration of suitable community-based or non-cancer specific services and challenges in matching people to the best available service for their level of needs.

To improve cancer survivors’ access to exercise supports, international experts have called for widespread implementation of triage and referral systems [9], which involve assessing a person to understand their needs, screening for any contra-indications or precautions regarding exercise, triaging to a suitable exercise pathway and making a referral where suitable. Triage and referral systems are underpinned by a triage algorithm, to standardise practice and ensure the person receives the right level of care for their individuals needs [10, 11]. As part of the Personalised Exercise Rehabilitation of Cancer Survivorship (PERCS) research project, we developed the PERCS triage and referral system which is designed to be applicable across a range of clinical contexts and to best integrate the local healthcare and exercise ecosystem [12].

Between January and August 2023, we delivered a pilot pre-post design study to understand the feasibility of implementing the PERCS triage and referral system in a clinical setting [12, 13]. Through a physiotherapy-led cancer rehabilitation clinic in a cancer specialist centre, we evaluated the PERCS triage and referral system, providing personalised recommendations to help participants improve their physical activity, including onwards referrals where appropriate. Participants were invited to attend a follow-up appointment at 12 weeks post-triage to review progress and provide feedback on the system. Results from the quantitative analysis published elsewhere [13] show that 90% of participants required support to become more physically active and that 66% of these patients could be referred to community exercise programmes. At the 12-week assessment, adherence to physical activity guidelines increased for aerobic (44% to 83%) and resistance (13% to 67%) exercise. Improvements were observed in hand grip strength (*x̄* 2.59 kg, *p* = 0.001), 30-s sit-to-stand (*x̄* + 3.7stands, *p* < 0.001) and 6-min walk test (*x̄* + 37.3 m, *p* = 0.001). A crucial additional stage of implementation analysis is consultation with participants to understand their experiences and the impact of the service, and to help us identify areas of improvement to ultimately deliver services which best meet patient needs [14, 15]. This qualitative study aimed to understand patient experiences of attending a physiotherapy-led triage and referral clinic.

## Methods

### Study design and ethical approval

This qualitative study used semi-structured interviews to gather detailed descriptions of participant experiences and perceptions of implementation of the PERCS triage and referral system. Approval was obtained from the Research and Innovation Office at St James’s Hospital (project number 7506). Ethical approval for this study was granted on 16th November 2023 by the St James’s Hospital (SJH)/Tallaght University Hospital (TUH) Joint Research Ethics Committee. This study was registered on ClinicalTrials.gov, on 21st October 2022 (registration number: NCT05615285) and was performed according to the Declaration of Helsinki. Findings are reported as per the COnsolidated criteria for REporting Qualitative research (COREQ) checklist [16].

### PERCS study context

**Fig. 1:**
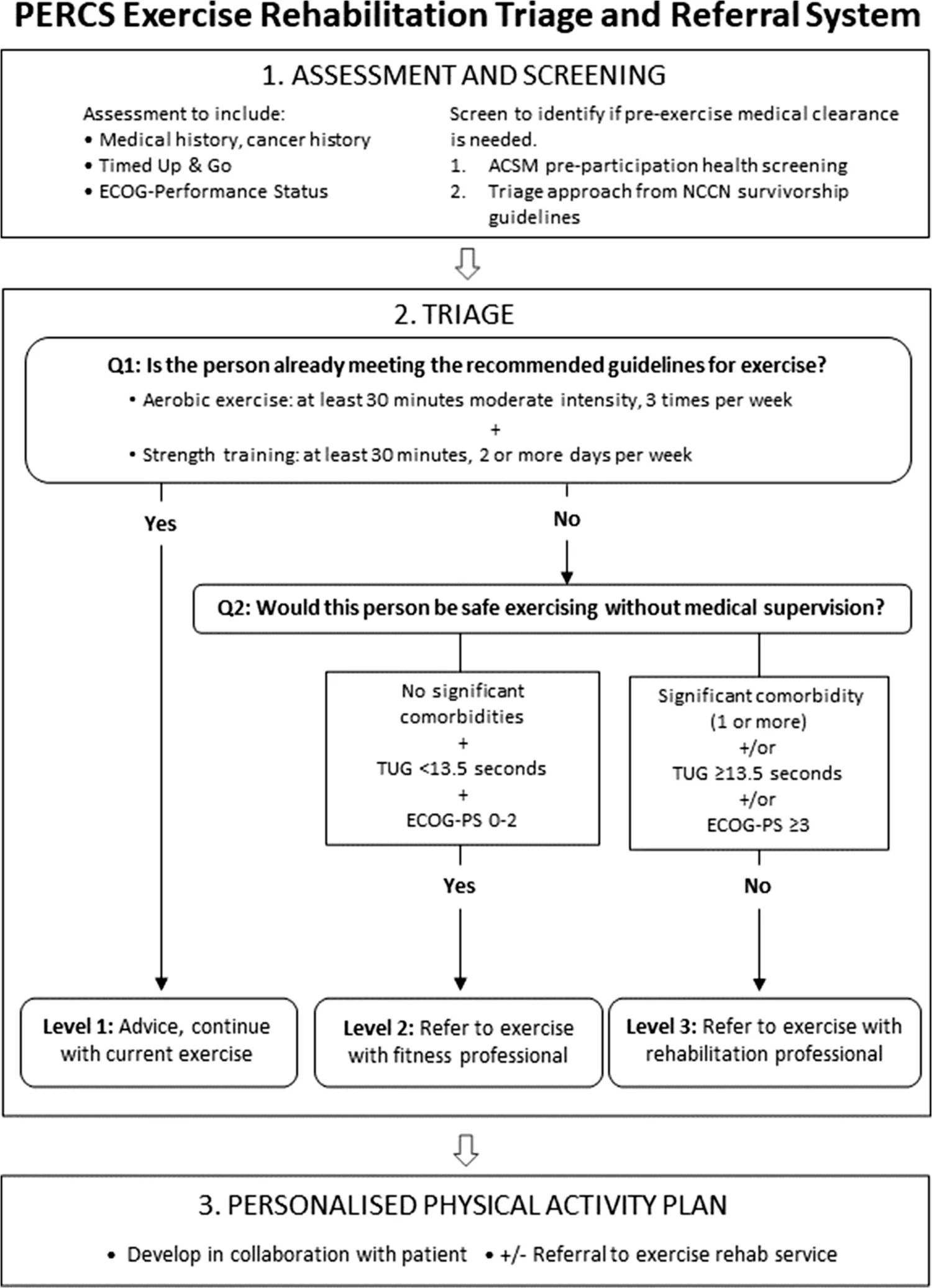
PERCS triage and referral system

This qualitative study was performed as part of the implementation evaluation of the PERCS triage and referral system for which the protocol [12] and the main findings [13] are fully reported elsewhere. The PERCS triage and referral system comprises patient assessment, triage and onward referral to one of the following three pathways (‘levels’) aligned with exercise and rehabilitation needs (Fig. 1):

- Level 1—meeting physical activity (PA) guidelines: encourage, advise and refer to www.cancerrehabilitation.ie.
- Level 2—not meeting PA guidelines, no medical comorbidities or rehabilitation impairments: refer to community exercise programme.
- Level 3—not meeting PA guidelines, presence of medical comorbidities or rehabilitation impairments: refer to physiotherapy/rehabilitation.

The in-person triage assessment was conducted in the context of a physiotherapy-led cancer rehabilitation clinical. This assessment included an assessment of habitual physical activity levels [9], past medical history underpinned by the National Comprehensive Cancer Network (NCCN) Survivorship Guidelines [5, 17], falls risk assessment using the Timed Up & Go (TUG) [18] and assessment of performance status using the Eastern Cooperative Oncology Group Performance-Status (ECOG-PS) [19]. Two *triage questions*, one informed by physical activity levels and one informed by comorbidity status, guide the user to one of three recommended pathways (‘triage levels’) for exercise rehabilitation advice and onward referral (Fig. 1) [5, 9]. *Triage outcomes* allocated participants to one of three evidence informed pathways (Table 1).

**Table 1:** Participant characteristics

Characteristic	Value
Age, mean (SD*), years	65 (11.62)
Sex, *n* (%) M F	15 (57.6) 11 (42.3)
Employment status, *n* (%) Retired Employed full time Stay at home parent Unable to work Unemployed Employed part time	14 (53.8) 4 (15.4) 3 (11.5) 3 (11.5) 1 (3.8) 1 (3.8)
Education, *n* (%) Primary Secondary Bachelor’s Master’s/higher diploma Professional accreditation Advanced certificate	2 (7.7) 13 (50) 3 (11.5) 6 (23.1) 1 (3.8) 1 (3.8)
Location, *n* (%) Urban Rural Suburban	11 (42.3) 8 (30.8) 7 (26.9)
Self-report to have a disability, *n* (%) Y N	15 (57.7) 11 (42.3)
Carer for other adult or child, n (%) Y N	7 (26.9) 19 (73.1)
English as first language, *n* (%) Y N	25 (96.1) 1 (3.9)
Cancer history	
Cancer type, *n* (%) Lung Colorectal Head and neck Upper gastro-intestinal Breast Gynaecological Thyroid	8 (30.8) 7 (26.9) 4 (15.4) 3 (11.5) 2 (7.7) 1 (3.8) 1 (3.8)
Months since diagnosis, mean (SD)	28.5 (6)
Months since last treatment, mean (SD)	22 (6.9)
PERCS triage outcome	
Triage level,*n* (%) 1: Meeting physical activity guidelines 2: Exercise with fitness professional 3: Exercise with healthcare professional	3 (11.5) 18 (69.2) 5 (19.2)

SD standard deviation

A 12-week exercise plan was created collaboratively between the physiotherapist and participant, and participants were invited to reassessment after 12 weeks. The process was supported by goal setting, a user-friendly 12-week exercise diary, a 1-week follow-up call and invitations to group psychosocial wellbeing seminars. Any membership costs incurred by participants associated with exercise referrals were covered by the research study. The PERCS system along with the evaluation methodology is described in full elsewhere [12]. A quantitative evaluation of the system involved a feasibility assessment examining recruitment and sample characteristics, evaluation of the triage and referral system data and outcome measures and evaluation of acceptability has been published elsewhere [13]. The assessment battery of physical activity, physical function and broader functional performance impairments included the International physical activity questionnaire (short-form), the Health Behaviour and States of change Questionnaire, the self-efficacy for exercise scale, hand grip strength, 30-s sit to stand test, body composition and the 6-min walk test. Here, we present qualitative findings from that evaluation.

### Participants and recruitment

Participants who had completed adjuvant chemotherapy and/or radiotherapy in St James’s Hospital between March 2020 and May 2022 were at least 6 weeks post-surgery, did not show signs of recurrent or metastatic disease at the time of enrolment, were aged > 18 years and were able to provide written informed consent were eligible to participate in the programme evaluation of the PERCS triage and referral system. Out of 64 participants enrolled to attend the PERCS triage and referral cancer rehabilitation clinic, purposive sampling was used to recruit participants representing a range of ages, cancer types, triage groups, physical activity levels and socioeconomic backgrounds. Participants who, when completing the PERCS informed consent form, provided written consent to take part in a semi-structured interview were invited to attend an interview at their final appointment in the PERCS clinic. Recruitment continued until data saturation was achieved.

### Development of interview guide

The interview guide was developed using the Consolidated Framework for Implementation Research (CFIR) [20]. CFIR is an implementation framework and consists of 37 constructs which are associated with successful implementation. By analysing an intervention against these constructs, the researcher can better understand the reasons why implementation was or was not successful [21]. CFIR constructs are clustered under five domains: intervention characteristics, outer setting, inner setting, characteristics of the individuals involved and the process of implementation. PERCS research team used the online CFIR interview guide development tool (https://cfirguide.org/guide/app/#/) to build a customised interview guide based on the CFIR constructs that are the focus of this implementation evaluation. Supplementary file 1 outlines the interview guide mapped against CFIR domains. No PERCS interview questions are mapped against the ‘process of implementation’ domain, as the four constructs in this domain (‘planning’, ‘engaging’, ‘executing’, ‘reflecting and evaluating’) are targeted to non-participant/user stakeholders, e.g. implementation leaders and opinion leaders. Interviews were digitally audio-recorded and transcribed verbatim. Transcripts were not returned to participants for review. Interview data was deductively analysed using pre-identified codes related to CFIR constructs, while also allowing for identification of new codes from the dataset [22, 23]. The CFIR provides a menu of constructs arranged across five domains that can be used in a range of applications. It is a practical theory-based guide for systematically assessing potential barriers and facilitators to guide tailoring of implementation strategies and adaptations for the innovation being implemented and/or explain outcomes.

### Data collection and analysis

Semi-structured interviews were conducted face-to-face, by telephone or through videoconferencing as convenient for the participant. Interviews were conducted by one of two female researchers: (1) EG a physiotherapist and Associate Professor in Cancer Rehabilitation and Survivorship, and the Principal Investigator of the PERCS study; (2) SG an oncology physiotherapist and researcher and (3) HC a research assistant in the Discipline of Occupational Therapy. EG and SG were introduced to participants by LB after the follow-up assessment. Interviews were transcribed, pseudonymised and coded using reflexive thematic analysis and deductive coding approach based on the CFIR constructs. Transcripts were coded independently by two researchers using the NVIVO 2.0 qualitative data analysis software (QSR International, Melbourne, Australia). Following a period of data familiarization, LB and MK independently deductively coded the data under the headings of the relevant CFIR framework. LB and MK then reviewed their findings to confirm high levels of agreement and to discuss any ambiguity or uncertainty related to specific codes or quotes. Through studying the quotes within each code and with a high level of familiarity with the overall dataset, both researchers discussed and agreed upon the overall findings for each CFIR construct and the most appropriate quotes were then selected.

### Investigator bias

The semi-structured interview guide was underpinned by the CFIR framework and reviewed iteratively in consultation with a multidisciplinary team to minimize bias in question design. Most interviews were completed by either SG or HC, neither of whom had any involvement in PERCS and therefore minimizing potential bias. The remaining interviews were conducted by the principal investigator EG, who was not involved in patient assessments, but as had to be very aware of not allowing her own bias to influence questioning. Any potential biases were managed by adhering to the interview script. Data analysis was conducted by LB, who also acted as the PERCS physiotherapist, and therefore had to manage her own biases through deductive coding to the CRIF framework. The second data analyst, MK, was not involved in PERCS did not know any of the participants and was not invested in the study outcomes, therefore analyzing through a completely neutral lens.

## Results

Twenty-six participants in the PERCS study were interviewed (24 in-person, two via telephone). Interviews lasted between 9 and 23 min. Participant characteristics are presented in Table 1. A summary of the CFIR constructs and related codes are presented in Table 2.

**Table 2:** Overview of CFIR domains, constructs and related codes

CFIR Domain	CFIR Construct	Codes
Outer setting	Patient needs and resources – Perceived Needs – Barriers and Facilitators – Participant Satisfaction and Feedback	Inadequate support during recovery Unmet physical and psychosocial needs A need for a personalised evaluation by a healthcare professional Practical issues create barriers to attendance Family support and a sense of accountability facilitate engagement Physical and psychosocial benefits, increased activity levels Participants recommend clinic to other cancer survivors
Intervention characteristics	Evidence strength and quality	PERCS aligns with participants understanding of benefits of exercise Measuring outcomes is valued
	Relative advantage	Few other services to compare to Advantage of personalised approach
	Design quality and packaging	Easy to follow and comprehensive Website often not required
	Cost	Mixed responses to costs, determined by income and spending priorities A worthwhile investment for health service
Inner setting	Implementation climate	Individually receptive to PERCS Value for health service lies in long-term benefits
	Tension for change	Emphatic that improved exercise rehabilitation services needed Contrast with rehabilitation in other clinical specialties Intolerable financial and health costs of not improving rehabilitation
Characteristics of individuals	Self-efficacy	Varied levels of self-efficacy to exercise Influenced by experience of exercise and barriers faced Knowledge improves self-efficacy
	Individual stage of change	Varied stages of change Shifts in stage of change observed

CFIR consolidated framework for implementation research, PERCS personalised exercise rehabilitation for cancer survivorship

### Outer setting

#### Patient needs and resources: perceived needs

Participants reported receiving little to no support after their cancer treatment finished and often felt alone during their recovery.

“There was no place where we can go and ask or talk about how you’re getting on over here” P06

“I just came home from hospital and I was on my own” P18

Across all patient demographics and cancer types, participants experienced physical and psychosocial rehabilitation needs. Sometimes they felt not listened to, or even dismissed, when they raised concerns about treatment side effects.

“I had issues with my bladder, and it was stopping me going out walking… when I told them in the hospital, it was like they’re really not interested” P65

Many participants wanted to speak with a specialist healthcare professional for individualised advice but were unsure of where to go.

“I wouldn’t have felt confident just going to see some random physio because of my medical needs. I would have felt ‘hold on, are these really qualified?’” P53

#### Patient needs and resources: barriers and facilitators

Practical issues, such as transport, parking, time of day and location had an important influence on the accessibility of PERCS clinic, as well as the local exercise programmes.

“The biggest drawback was getting here…and the parking is always full, that’s a bit tricky.” P09

Support and encouragement from family members was a key facilitator for a number of participants.

“when I was invited up for it…it was more the wife pushing me into it, really. She said, ‘Look, what have you got to lose?’ And I’m glad she did.” P30

The follow-up appointment and exercise diaries provided a sense of accountability and encouraged some participants to engage in exercise when they felt low in motivation.

“Whether you want to go out or not, you push yourself, you know, ‘Oh, gosh, [researcher] will be waiting for me.’ She gave me the diary just to fill in each day…it just pushed me to go out and walk.” P34

#### Patient needs and resources: participant satisfaction and feedback

Participants reported being more physically active since attending the PERCS clinic. Some established new habits and some began to prioritise exercise in a way they had not before. Through taking part in PERCS, participants experienced physical, mental and functional benefits.

*“I realised I got into the habit of going, ‘I can’t do that, I can’t lift that.’ I’m waiting for someone else to come and rescue the situation. But now I’m like, ‘Oh, back off. I can do it myself.’…it’s given me such a new outlook and just more confidence over the last 12 weeks.” P17.*

“The fogginess is cleared from my mind. I find the more alert, I’m less forgetful.” P53

“I turned 80 this week…So I feel great. And I’m delighted with how I feel.” P18

Some participants were emphatic that they had experienced very substantial improvements in their quality of life:

“I’m able to go for a walk now without having to make sure that there’s a bathroom there…that for me is a huge, huge impact on my life.” P65

“It’s basically an 80% improvement on my health and my mindset is completely different as well.” P30

Receiving feedback at the follow-up appointment was motivational and rewarding, and gave some sense of pride.

Overall, participants were happy they attended PERCS clinic and engaged in the physical activity recommendations, and would highly recommend it to other cancer survivors:

“All I was thinking is that anything has got to be better than where I was, and I didn’t realise how good it was going to be. I find it very hard to understand why anyone wouldn’t benefit from it, I really do.” P57

### Intervention characteristics

#### Evidence strength and quality

Participants expressed a newfound understanding of the evidence-based benefits of exercise after cancer, and they understood that PERCS was rooted in these principles:

“The whole approach of PERCS was very, very good. This idea that exercise is good for your body and your mind, which of course I knew instinctively anyway, but probably hadn’t articulated it to myself.” P15

Additionally, several participants expressed an appreciation for how validated outcome measures were used to measure baselines and demonstrate change:

“It was very well organized—setting the baseline, seeing where I am not only physically but also psychologically, which is critically important.” P60

#### Relative advantage

No other triage clinics were identified by participants, and most participants were unaware of any other cancer rehabilitation services prior to attending PERCS clinic. Some participants could mention one other exercise rehabilitation service, such as a class in a local community centre or cancer support centre, and then explained why these services were unsuitable for them:

“They were looking at like, physiotherapy or some kind of thing three times a week. Yeah. I just wouldn’t have find time to do that, even online.” P01

PERCS allowed participants to develop a personalised exercise programme that overcame these barriers:

“what I really liked about the PERCS was, in a way, it kind of was tailor made. So, ‘Listen, we’re going to suit your needs and look at what’s going to be good for you.’” P53

#### Design quality and packaging

All written materials were found to be easy to follow, comprehensive and well laid-out:

“Everything was there, it was clear, concise and I knew exactly what I was facing when I agreed to come on the programme.” P60

The associated website developed as part of PERCS research project was generally not used by participants. Some reported not being interested in an online resource, while others felt that their needs had been met by attending the PERCS clinic, plus or minus a local exercise programme, and therefore did not seek additional information.

#### Cost

Financial costs for participants included money spent on attending the clinic (e.g. travel and parking) as well as the potential costs of continuing any fee-paying exercise programme, after the PERCS-funded initial 12 weeks was complete. Participants had mixed feelings towards these expenses. For some, the costs were small and not an issue, and this group acknowledged that this would not be the case for everyone:

“I was able to [pay] but I would say that would be very prohibitive for a lot of people.” P04

For others, costs were an issue. One older participant described the careful thought process around undertaking this new expense:

“I’m retired now, I’ve only got a pension, that’s all I have…[the exercise programme] would cost me €10 a week, which is €40 a month….I’d be saying to myself, you know ‘another couple euro, I might be able to get a gym where I’d be—I could maybe get a swim as well.’” P18.

After seeing the benefits of exercise, some participants were more willing to spend money on it than they would have been before:

“Before, I wouldn’t have thought it was a priority expense, but now I’m definitely thinking, no, I have to, it is a priority. It’s my health, it’s what’s going to keep me going.” P17

Several participants discussed how PERCS is a worthwhile system for the health service to invest in, which would prove cost-effective in the long-term:

“I’m aware of how much the [Health Service Executive] spends in its budget and certainly it would be worth considering diverting some of that budget stream into the program without a doubt.” P15

### Inner setting

#### Implementation climate

On an individual level, participants felt that patients would be receptive towards the PERCS clinic, and they highly endorsed that every cancer survivor should have the opportunity to attend:

“I think everyone that has been over cancer survival should try their best to partake in this program.” P34

“I’ve told so many people about this because I just think that this should be offered to all cancer survivors.” P57

On a systems level, participants identified there could be reluctance due to initial costs of setting up a new service, but that it would be a wise investment:

“I would recommend: one, roll [PERCS] out; two, that it becomes a part of cancer treatment…of any cancer treatment. And it will save the health service millions and millions if they can invest in this program to help people.” P60.

#### Tension for change

There was a strong sentiment that improved exercise rehabilitation services are needed:

“It should be in every village and town in the country” P15

Some participants contrasted their cancer rehabilitation with their personal experiences in cardiovascular health care:>

“When I had my first heart attack, we were brought back straight away by the hospital into rehab and exercising every week, you know, and that stage, that’s 20 years ago!” P15

“If PERCS was there in Limerick, in Cork and Galway, and this is where you go once you’ve had cancer. You would get it if you had a heart attack.” P54

“If PERCS was there in Limerick, in Cork and Galway, and this is where you go once you’ve had cancer. You would get it if you had a heart attack.” P54

Participants were emphatic that changes in survivorship care are necessary, and that the downstream costs, both financial and health-related, of not providing rehabilitation were inexcusable:

“When somebody goes through what I’ve gone through, whether it’s a single mastectomy, whatever it is, there needs to be, 100%, something put in place, whether they like it or not. And it shouldn’t be an option. …People aren’t superhuman. [Rehabilitation] needs to be improved upon and it needs to be taken more seriously.” P37.

“It’s the same as investing in an alcohol program or no smoking program. This is just as important as prehab as your treatment.” P60

### Characteristics of individuals

#### Self-efficacy

When reflecting on their feelings prior to attending the PERCS clinic, participants reported varying level of self-efficacy towards engaging in exercise. Some felt very confident, while others were quite apprehensive. Levels of self-efficacy were frequently influenced by a participant’s fitness levels, their experiences of exercise, and the barriers they faced:

“I was confident probably because I felt I’ve always been fairly fit. Yeah. So, I wasn’t in any way apprehensive about coming into it.” P53

“It’s possible that I probably would have stuck to [an exercise programme] better if I had to actually go somewhere and do it rather than [exercise alone] at home.” P22

#### Individual state of change

Upon describing their exercise habits and attitudes prior to attending PERCS clinic, it could be seen that participants were in a variety of stages of change, including contemplation, preparation and maintenance.

“The timing of meeting [the researcher] was critical because I was taking no exercise at all and quite nervous about my own health situation.” P15

“I suppose I was quite fit coming in anyway and I would be conscious of kind of looking after myself and trying to put the effort in.“ P37

Many participants displayed a progression in state of change over the 12 weeks. They actively changed their exercise behaviours, developed sustainable activity habits and, for some, a new lease of life:

“I think I could manage [to continue exercising home]. And in actual fact, I think that’s what I would do.” P62

“Coming here was definitely worthwhile. And it sort of opened me up to who I could be, whereas at my age I was just sort of more or less feeling life was over.” P56

## Discussion

This study explored the factors that influenced implementation of the PERCS triage and referral system from a patient’s perspective. In physical activity programmes, patient engagement, participation and behaviour change are central to implementation success, and therefore understanding the patient experience of implementing the PERCS recommendations, will inform the future directions of this programme. Results show that exercise programming is a major unmet need within cancer survivorship pathways, and that this need could be addressed by personalised triage and referral systems such as PERCS. Participants reported high levels of satisfaction with all aspects of the intervention characteristics; however, sustainability could be impacted by financial cost. The opportunity to meet with a specialist physiotherapist was highly valued by participants across all triage levels and addressed multiple physical and psychosocial needs.

The PERCS triage and referral system is a three-staged process comprising individual patient assessment with an oncology physiotherapist, triage to one of three levels of care based on rehabilitation needs and creation of a 12-week personalised physical activity plan, including referral to a suitable exercise or rehabilitation service as appropriate. The system was supported by a website (www.cancerrehabilitation.ie) which hosts a national directory of exercise-based rehabilitation services suitable for cancer survivors and provides information about exercise and cancer rehabilitation. As an intervention, PERCS was well received by participants, was seen as an advantage over standard care, enabled patients to develop a better understanding of the role of exercise in cancer and the structure of the service was met with satisfaction. Financial cost however was highlighted as a potential barrier to sustained participation, particularly in community-based programmes (level 2). The mean cost for attending level 2 exercise services was €107.70, and these costs were covered by the research team as a requirement of the research ethics committee [13]. For some patients, this cost was not an issue or was perceived to be good value, whereas other patients were unsure if they could manage the expense themselves. In addition, the median cost of travel to the PERCS assessments alone was €9.40, ranging from €2 to €124. Travel burden is repeatedly cited as a primary barrier to participation in exercise-oncology trials [24, 25], and financial burden of travel and participation is a component of this concern. This is an important concern as the majority of participants within PERCS (67%) were triaged to level 2. Cancer rehabilitation triage and referral systems like PERCS, EXCEEDS [26] and ACE [10] apply a tailored approach in identifying the most suitable, accessible, community-based services for cancer survivors. This reduces travel burden on patients, and reserves healthcare-based services for those most in need of them, reducing unnecessary demand on these clinics. Triage and referral systems have potential to be high-value, cost-effective interventions, and future work needs to firmly establish their cost-effectiveness. This will demonstrate the need for, and benefit of, government and health service investments in exercise and cancer rehabilitation services.

Participants in this qualitative study were on average 28.5 months following their cancer diagnosis, were disease-free and had independently read and understood all trial participation documentation; participants therefore reflect a cohort who are medically well with considerable health literacy and willingness to engage with health services. However, even within this cohort, self-efficacy for exercise participation varied. Participants reported receiving specialist physiotherapy advice at their PERCS appointments that they did not feel was available to them, or had been provided to them, elsewhere. After receiving just one personalised assessment, most participants experienced changes in exercise behaviours and attitudes, and a progression in ‘state of change’ was observed, as well as perceived improvements in physical and psychosocial metrics [13]. These changes were also demonstrated in the objective measures reported elsewhere, where improvements in hand grip strength, sit-to-stands, 6-min walk test and major improvements in adherence to both aerobic (44% to 83%) and resistance (13% to 67%) exercise from baseline to 12 weeks [13] were observed. These results highlight the impact of a relatively simple, clinically pragmatic but personalised approach that focuses on reducing barriers, harnessing facilitators and supporting patients to find the right service to meet their needs. Further work should focus on examining the effectiveness of the PERCS system at different stages throughout the cancer trajectory.

The findings described in this paper are consistent with existing literature in demonstrating that cancer rehabilitation is typically not prioritised for patients in the context of survivorship care pathways and that patients as the key stakeholders believe that this situation needs to change [6, 7]. This project was funded in response to the COVID-19 pandemic and consequently recruited participants who were treated during the pandemic, which may have further limited access to services. There was a sense of frustration, astonishment and even anger at the lack of cancer rehabilitation services, both in participants’ own experiences, and what they perceive to be available on a national level. This may have been exacerbated by limited access to services during the pandemic and participants perceptions of the level of services locally and nationally may be different in the post-pandemic period. We have previously described inadequate levels of cancer rehabilitation services nationally [7], and unfortunately this problem is reflected internationally. There is much high-quality research into barriers to service development and how to implement recommended models of care [27, 28]. Participants drew a clear comparison between cardiac rehabilitation and cancer rehabilitation, a comparison which is frequently also drawn in the aforementioned research. One participant’s call for help because ‘people aren’t superhuman’ is particularly compelling as it paints a picture of an individual simply seeking compassion and help during a time of vulnerability.

Understanding how triage and referral systems like PERCS and others fit into healthcare systems is critical. PERCS aligns very closely with the National Exercise Referral Framework in Ireland which recognizes a tiered service provision structure for exercise in chronic disease that ranges from specialized to self-directed exercise [29]. Ireland has a growing number of government funded cancer rehabilitation services and availability of low-cost community exercise classes, particularly for people aged > 65 years. A proportion of the Irish population will be able to self-fund attendance at classes, as reflected in our findings. With growing economic inequality and health inequity [30], it is increasingly important that researchers and clinicians ensure that cost is not a barrier to engaging in health-related services. Nevertheless, this evaluation of PERCS was set in the context of a single-centre with relatively high resource available to support referral pathways, but it is unlikely that the same level of service is replicated across Ireland. PERCS 2.0, recently funded by the Health Research Board in Ireland (grant reference code ILP-HSR-2024–011, will investigate the issue of national implementation through the completion of two work streams. The first will examine the cost effectiveness of PERCS using a model-based simulation which will considering wider healthcare use for patients with cancer, the total costs of programme delivery and the incremental cost effectiveness of PERCS. This information, while directly relevant to the Irish healthcare context, may provide transferable information for other healthcare systems underpinned by different public or private healthcare funding models. The second workstream planned in PERCS 2.0 will establish an implementation strategy for PERCS, considering the barriers and facilitators to embedding PERCS into the cancer care pathways and identifying relevant, related individual implementation strategies to address these issues. This piece will refine how the triage pathways may be applied in a national context and while context-specific to Ireland, may provide valuable learnings for other healthcare systems.

A key strength of this study is that participants were purposively sampled to ensure representation across levels of rehabilitation need, national geography and gender, and therefore the experiences reported represent a wide range of cancer survivors. The interview guide was constructed to examine patient experiences through an implementation lens, underpinned by the CFIR framework; an updated version of CFIR is now available [27]. While participant numbers and representation are strong, only participants who returned for follow up assessment were recruited for interview, and data on those who did not return is not available, which may positively bias experiences and perceptions of the intervention. As presented in our quantitative evaluation [13], 46/64 (72%) of participants returned for re-assessment at 12 weeks. Consequently, the results are mainly positive and provide limited insights into barriers or challenges for the 30% of participants who did not return. Future research should aim to include provision for conducting interviews with participants who drop-out where ethically possible. Globally, it is difficult to capture rich data from those who do not recruit to or who are not retained in exercise oncology or cancer rehabilitation programmes, thereby often limiting our ability to capture insights into the challenges that exist for these participants [24, 25].

## Conclusions

In conclusion, the PERCS triage and referral system was well received by participants and delivered considerable benefits to patients. Participants felt strongly that exercise and rehabilitation services are inadequate in current cancer care pathways, and the PERCS system addresses this service gap. High levels of participant satisfaction with the implementation processes underpinning PERCs reinforces the potential for triage and referral systems to successfully support implementation of cancer rehabilitation and exercise pathways into cancer care. Self-efficacy to engage in exercise independently was varied, and specialist assessment, triage and referral by the oncology physiotherapist was a key factor driving participant satisfaction.

## Supplementary Information

Below is the link to the electronic supplementary material.

ESM 1 (DOCX 30.0 KB)

## Data Availability

Data is available on request to the corresponding author.

## References

[1] Nekhlyudov L, Campbell GB, Schmitz KH, Brooks GA, Kumar AJ, Ganz PA, Von Ah D. Cancer-related impairments and functional limitations among long-term cancer survivors: gaps and opportunities for clinical practice. Cancer. 2022;128(2):222–9. 10.1002/cncr.33913 PMC9292035 34529268

[2] Flores AM, Shah M, Bedjeti K, Franklin PD, Peipert JD, Garcia SF, Lancki N, Webster KA, O’Connor M, Cella D. Risk of significant functional impairment across cancer diagnosis and care continuum. Cancer. 2024;131(1): e35571. 10.1002/cncr.35571. PMC11694161 39297349

[3] Elliott J, Fallows A, Staetsky L, Smith PW, Foster CL, Maher EJ, Corner J. The health and well-being of cancer survivors in the UK: findings from a population-based survey. British J Cancer. 2011;105 Suppl 1(Suppl 1):S11-20. 10.1038/bjc.2011.418 PMC3251954 22048028

[4] Aaronson NK, Mattioli V, Minton O, Weis J, Johansen C, Dalton SO, Verdonck-de Leeuw IM, Stein KD, Alfano CM, Mehnert A, et al. Beyond treatment - psychosocial and behavioural issues in cancer survivorship research and practice. EJC Suppl. 2014;12(1):54–64. 10.1016/j.ejcsup.2014.03.005 PMC4250535 26217166

[5] Campbell KL, Winters-Stone KM, Wiskemann J, May AM, Schwartz AL, Courneya KS, Zucker DS, Matthews CE, Ligibel JA, Gerber LH, et al. Exercise guidelines for cancer survivors: consensus statement from international multidisciplinary roundtable. Med Sci Sports Exerc. 2019;51(11):2375–90. 10.1249/MSS.0000000000002116 PMC8576825 31626055

[6] Doughty HC, Hill RA, Riley A, Midgley AW, Patterson JM, Boddy LM, Rogers SN, Maden M, Williams NH. Barriers to and facilitators of physical activity in adults living with and beyond cancer, with special emphasis on head and neck cancer: a systematic review of qualitative and mixed methods studies. Support Care Cancer. 2023;31(8):471. 10.1007/s00520-023-07925-x PMC10352410 37458858

[7] Brennan L, Sheill G, O’Neill L, O’Connor L, Smyth E, Guinan E. Physical therapists in oncology settings: experiences in delivering cancer rehabilitation services, barriers to care, and service development needs. Phys Ther. 2022;102(3):pzab287. 10.1093/ptj/pzab287 PMC8887570 35084029

[8] Kennedy MA, Bayes S, Newton RU, Zissiadis Y, Spry NA, Taaffe DR, Hart NH, Galvão DA. Implementation barriers to integrating exercise as medicine in oncology: an ecological scoping review. J Cancer Surviv. 2022;16(4):865–81. 10.1007/s11764-021-01080-0 PMC9300485 34510366

[9] Schmitz KH, Campbell AM, Stuiver MM, Pinto BM, Schwartz AL, Morris GS, Ligibel JA, Cheville A, Galvão DA, Alfano CM, et al. Exercise is medicine in oncology: engaging clinicians to help patients move through cancer. CA: A Cancer J Clin. 2019;69(6):468–84. 10.3322/caac.21579 PMC7896280 31617590

[10] Capozzi LC, Daun JT, Francis GJ, de Guzman WM, Urgoiti GR, Langelier D, Culos-Reed N. Feasibility and implementation of an oncology rehabilitation triage clinic: assessing rehabilitation, exercise need, and triage pathways within the alberta cancer exercise-neuro-oncology study. Curr Oncol. 2023;30(7):6220–45. 10.3390/curroncol30070461 PMC10377964 37504321

[11] Covington KR, Marshall T, Campbell G, Williams GR, Fu JB, Kendig TD, Howe N, Alfano CM, Pergolotti M. Development of the exercise in cancer evaluation and decision support (EXCEEDS) algorithm. Support Care Cancer. 2021;29(11):6469–80. 10.1007/s00520-021-06208-7 PMC8525648 33900458

[12] Brennan L, Sheill G, Collier S, Browne P, Donohoe CL, O’Neill L, Hussey J, Guinan EM. Personalised exercise rehabilitation in cancer survivorship: the PERCS triage and referral system study protocol. BMC Cancer. 2024;24:517. 10.1186/s12885-024-12266-x PMC11040825 38654198

[13] Brennan L, Sheill G, Collier S, Browne P, Donohoe C, Guinan E. Personalised exercise rehabilitation in cancer survivorship: findings from a triage and referral feasibility study. J Cancer Surviv. 2024. 10.1007/s11764-024-01684-2. 39528780

[14] Wensing M, Elwyn G. Methods for incorporating patients’ views in health care. BMJ. 2003;326(7394):877–9. 10.1136/bmj.326.7394.877 PMC1125777 12702627

[15] Duarte J, Guerra A. User-centered healthcare design. Procedia Comput Sci. 2012;14:189–97.

[16] Tong A, Sainsbury P, Craig J. Consolidated criteria for reporting qualitative research (COREQ): a 32-item checklist for interviews and focus groups. Int J Qual Health Care. 2007;19(6):349–57. 10.1093/intqhc/mzm042 17872937

[17] National Comprehensive Cancer Network. NCCN clinical practice guidelines in oncology - Survivorship. 2018; Version 2. 2018.

[18] Podsiadlo D, Richardson S. The timed “Up & Go”: a test of basic functional mobility for frail elderly persons. J Am Geriatr Soc. 1991;39(2):142–8. 10.1111/j.1532-5415.1991.tb01616.x 1991946

[19] Azam F, Latif MF, Farooq A, Tirmazy SH, AlShahrani S, Bashir S, Bukhari N. Performance status assessment by using ECOG (Eastern Cooperative Oncology Group) score for cancer patients by oncology healthcare professionals. Case Rep Oncol. 2019;12(3):728–36. 10.1159/000503095 PMC6792426 31616281

[20] Damschroder LJ, Aron DC, Keith RE, Kirsh SR, Alexander JA, Lowery JC. Fostering implementation of health services research findings into practice: a consolidated framework for advancing implementation science. Implement Sci. 2009;4: 50. 10.1186/1748-5908-4-50 PMC2736161 19664226

[21] King DK, Shoup JA, Raebel MA, Anderson CB, Wagner NM, Ritzwoller DP, Bender BG. Planning for implementation success using RE-AIM and CFIR frameworks: a qualitative study. Front Public Health. 2020;8: 59. 10.3389/fpubh.2020.00059 PMC7063029 32195217

[22] Nevedal AL, Reardon CM, Opra Widerquist MA, Jackson GL, Cutrona SL, White BS, Damschroder LJ. Rapid versus traditional qualitative analysis using the Consolidated Framework for Implementation Research (CFIR). Implement Sci. 2021;16(1):67. 10.1186/s13012-021-01111-5 PMC8252308 34215286

[23] Damschroder LJ, Lowery JC. Evaluation of a large-scale weight management program using the consolidated framework for implementation research (CFIR). Implement Sci. 2013;8: 51. 10.1186/1748-5908-8-51 PMC3656778 23663819

[24] Kearney N, Connolly D, Begic S, Mockler D, Guinan E. Feasibility metrics of exercise interventions during chemotherapy: a systematic review. Crit Rev Oncol Hematol. 2024;195:104272. 10.1016/j.critrevonc.2024.104272 38272152

[25] Reynolds SA, O’Connor L, McGee A, Kilcoyne AQ, Connolly A, Mockler D, Guinan E, O’Neill L. Recruitment rates and strategies in exercise trials in cancer survivorship: a systematic review. J Cancer Surviv. 2023;18(4):1233-1242. 10.1007/s11764-023-01363-8 PMC11324688 37022641

[26] Wood KC, Pergolotti M, Marshall T, Leach HJ, Sharp JL, Campbell G, Williams GR, Fu JB, Kendig TD, Howe N, et al. Usability, acceptability, and implementation strategies for the Exercise in Cancer Evaluation and Decision Support (EXCEEDS) algorithm: a Delphi study. Support Care Cancer. 2022;30(9):7407–18. 10.1007/s00520-022-07164-6 35614154

[27] Segal R, Zwaal C, Green E, Tomasone JR, Loblaw A, Petrella T. Exercise for people with cancer: a clinical practice guideline. Curr Oncol. 2017;24(1):40–6. 10.3747/co.24.3376 PMC5330628 28270724

[28] Wolin KY, Schwartz AL, Matthews CE, Courneya KS, Schmitz KH. Implementing the exercise guidelines for cancer survivors. J Support Oncol. 2012;10(5):171–7. 10.1016/j.suponc.2012.02.001 PMC3543866 22579268

[29] Woods C, McCaffrey N, Furlong B, Fitzsimons-D’Arcy L, Murphy M, Harrison M, Glynn L, O’Riordan J, O’Neill B, Jennings S, Peppard C. The national exercise referral framework. Health and wellbeing division, health service executive. Dublin; 2016.

[30] Cancer Pulse Tracking Inequalities in Cancer. Ireland: Country Report; 2024. https://www.europeancancer.org/pulse.

