# Supplementary material for: Patient experiences of participating in a cancer rehabilitation triage and referral system in the Irish healthcare system
Source: J Cancer Surviv. 2025 Mar 27;20(Suppl 1):28–39. doi: 10.1007/s11764-025-01785-6 (PMC13676489; doi:10.1007/s11764-025-01785-6)
Supplement: Supplementary file 1 [file 11764_2025_1785_MOESM1_ESM.docx]

**PERCS Interview Guide**

**Introductory questions**

IQ1. Please describe your overall experience of the PERCS service.

IQ2. What exercise advice / referral were you given by the physiotherapist?

- Did you follow this advice / attend the service?
- If so, can you tell me about this? If not, why?

| CFIR construct | CFIR-generated question | PERCS interview question |
| --- | --- | --- |
| Intervention Characteristics  Evidence Strength & Quality | What do influential stakeholders think of the intervention? | What are your overall thoughts about the PERCS system?  What did you like/not like about PERCS? |
| Intervention Characteristics  Relative Advantage | Is there another intervention that people would rather implement?  Can you describe that intervention?  Why would people prefer the alternative? | Are you aware of other systems/services which support people to become more active after cancer?  If so, please describe  What do you think are the advantages of this alternative system? |
| Intervention Characteristics  Design Quality & Packaging | What is your perception of the quality of the supporting materials, packaging, and bundling of the intervention for implementation? Why? | What are your thoughts on the quality of the written materials used in PERCS (PIL, diary)?   - Are there any changes you would recommend to these materials?   Did you visit the PERCS webpage?   - If so, what are your thoughts on it? - What did you find helpful / not helpful? - Are there any changes you would recommend? |
|  | What supports, such as online resources, marketing materials, or a toolkit, are available to help you implement and use the intervention?  How do you access these materials? |  |
| Intervention Characteristics  Cost | What costs will be incurred to implement the intervention? | Did it cost you anything (financially) to take part in PERCS? If so, can you describe the costs? |
| Outer Setting  Patient Needs & Resources | To what extent is staff aware of the needs and preferences of the individuals being served by your organization?  How "in touch" are staff and leadership with the individuals served by your organization? | Do you feel that the PERCS system and the PERCS team understand the needs and preferences of people who have had cancer treatment?  Do you think the PERCS system successfully meets the needs of people who have had cancer treatment? How? |
|  | How well do you think the intervention will meet the needs of the individuals served by your organization?  In what ways will the intervention meet their needs? E.g. improved access to services? Reduced wait times? Help with self-management? Reduced travel time and expense? | Do you think the PERCS system successfully meets the needs of people who have had cancer treatment? How? |
|  | How do you think the individuals served by your organization will respond to the intervention? | In general, how do you think people who have had cancer would respond to the PERCS system? |
|  | What barriers will the individuals served by your organization face to participating in the intervention? | Can you think of any reason or situation where someone would find it difficult to take part in the PERCS system?   - Was it ever difficult for you? - Is there any way we can make it easier for people to attend? |
|  | Have you elicited information from participants regarding their experiences with the intervention?  What are their perceptions of the intervention?  Can you describe what kind of specific information you have heard? | Please describe your overall experience of the PERCS service. (IQ1)  What are your overall thoughts about the PERCS system?  What did you like/not like about PERCS? (Intervention characteristics) |
| Inner Setting  Implementation Climate  Tension for Change | Is there a strong need for this intervention?  Why or why not?  Do others see a need for the intervention?  How essential is this intervention to meet the needs of the individuals served by your organization or other organizational goals and objectives? |  |
|  | How do people feel about current programs/practices/process that are available related to the intervention?  To what extent do current programs fail to meet existing needs? Will the intervention meet these needs?  How will the intervention fill current gaps? | Are you aware of other systems/services which support people to become more active after cancer?  If so, please describe. (Intervention characteristics)  Do you think the other systems/services fully meet the needs of people after cancer?   - If not, can you describe how? - How does PERCS compare to that system / does PERCS address this ‘unmet need’? |
| Characteristics of Individuals  Self-efficacy | How confident are you that you will be able to use the intervention?  What gives you that level of confidence (or lack of confidence)? | Before you attended your first assessment, how confident did you feel that you would be able to use the PERCS system?  Why did you feel this way? |
| Characteristics of Individuals  Individual State of Change | *Explore which level the individual is at using Rogers' (or Porchaska's Stages of Change) as a guide:*  How prepared are you to use the intervention?  Knowledge stage (Precontemplation) - knowledge of key aspects of the intervention  Persuasion stage (Contemplation) - likes the intervention, discusses it with others, buys into it, has a positive view  Decision stage (Preparation) - intends to seek additional information and try it  Implementation stage (Action) - acquires additional information, uses intervention regularly, and has continued use  Confirmation stage (Maintenance) - recognizes benefits, has integrated the intervention into routines, promotes use to others | Data collected at initial assessment: Health Behaviour and Stages of Change Questionnaire |
